# Validation of a Radiography-Based Quantification Designed to Longitudinally Monitor Soft Tissue Calcification in Skeletal Muscle

**DOI:** 10.1371/journal.pone.0159624

**Published:** 2016-07-20

**Authors:** Stephanie N. Moore, Gregory D. Hawley, Emily N. Smith, Nicholas A. Mignemi, Rivka C. Ihejirika, Masato Yuasa, Justin M. M. Cates, Xulei Liu, Jonathan G. Schoenecker

**Affiliations:** 1 Department of Orthopaedics and Rehabilitation, Vanderbilt University Medical Center, Medical Center East, South Tower, Suite 4200, 1215 21th Avenue South, Nashville TN, 37232, United States of America; 2 School of Medicine, Vanderbilt University, 2215 Garland Ave, Light Hall, Room 215, Nashville, TN, 37232, United States of America; 3 Department of Pathology, Microbiology, and Immunology, Vanderbilt University Medical Center, 1161 21st Ave S, Nashville, TN, 37232, United States of America; 4 Department of Biostatistics, Vanderbilt University, 2525 West End, Ste 11000, Nashville, TN, 37203, United States of America; 5 Department of Pediatrics, Vanderbilt University Medical Center, 4202 Doctor’s Office Tower, 2200 Children’s Way, Nashville, TN, 37232, United States of America; 6 Department of Pharmacology, Vanderbilt University, 2200 Pierce Ave, Robinson Research Building Nashville, TN, 37232, United States of America; ACTREC, Tata Memorial Centre, INDIA

## Abstract

**Introduction:**

Soft tissue calcification, including both dystrophic calcification and heterotopic ossification, may occur following injury. These lesions have variable fates as they are either resorbed or persist. Persistent soft tissue calcification may result in chronic inflammation and/or loss of function of that soft tissue. The molecular mechanisms that result in the development and maturation of calcifications are uncertain. As a result, directed therapies that prevent or resorb soft tissue calcifications remain largely unsuccessful. Animal models of post-traumatic soft tissue calcification that allow for cost-effective, serial analysis of an individual animal over time are necessary to derive and test novel therapies. We have determined that a cardiotoxin-induced injury of the muscles in the posterior compartment of the lower extremity represents a useful model in which soft tissue calcification develops remote from adjacent bones, thereby allowing for serial analysis by plain radiography. The purpose of the study was to design and validate a method for quantifying soft tissue calcifications in mice longitudinally using plain radiographic techniques and an ordinal scoring system.

**Methods:**

Muscle injury was induced by injecting cardiotoxin into the posterior compartment of the lower extremity in mice susceptible to developing soft tissue calcification. Seven days following injury, radiographs were obtained under anesthesia. Multiple researchers applied methods designed to standardize post-image processing of digital radiographs (N = 4) and quantify soft tissue calcification (N = 6) in these images using an ordinal scoring system. Inter- and intra-observer agreement for both post-image processing and the scoring system used was assessed using weighted kappa statistics. Soft tissue calcification quantifications by the ordinal scale were compared to mineral volume measurements (threshold 450.7mgHA/cm^3^) determined by μCT. Finally, sample-size calculations necessary to discriminate between a 25%, 50%, 75%, and 100% difference in STiCSS score 7 days following burn/CTX induced muscle injury were determined.

**Results:**

Precision analysis demonstrated substantial to good agreement for both post-image processing (κ = 0.73 to 0.90) and scoring (κ = 0.88 to 0.93), with low inter- and intra-observer variability. Additionally, there was a strong correlation in quantification of soft tissue calcification between the ordinal system and by mineral volume quantification by μCT (Spearman r = 0.83 to 0.89). The ordinal scoring system reliably quantified soft tissue calcification in a burn/CTX-induced soft tissue calcification model compared to non-injured controls (Mann-Whitney rank test: *P* = 0.0002, ***). Sample size calculations revealed that 6 mice per group would be required to detect a 50% difference in STiCSS score with a power of 0.8. Finally, the STiCSS was demonstrated to reliably quantify soft tissue calcification [dystrophic calcification and heterotopic ossification] by radiographic analysis, independent of the histopathological state of the mineralization.

**Conclusions:**

Radiographic analysis can discriminate muscle injury-induced soft tissue calcification from adjacent bone and follow its clinical course over time without requiring the sacrifice of the animal. While the STiCSS cannot identify the specific type of soft tissue calcification present, it is still a useful and valid method by which to quantify the degree of soft tissue calcification. This methodology allows for longitudinal measurements of soft tissue calcification in a single animal, which is relatively less expensive, less time-consuming, and exposes the animal to less radiation than *in vivo* μCT. Therefore, this high-throughput, longitudinal analytic method for quantifying soft tissue calcification is a viable alternative for the study of soft tissue calcification.

## Introduction

Soft tissue calcification after injury can result in significant patient morbidity including pain and joint dysfunction, which can ultimately lead to loss of limb function and subsequently a patient’s ability to perform activities of daily living [1]. Soft tissue calcification includes two histopathologically distinct forms; dystrophic calcification [2, 3] and heterotopic ossification [4]. While dystrophic calcification is defined as amorphous deposits of calcium phosphate in damaged soft tissues, heterotopic ossification is the formation of mature, mineralized bone tissue Although the histopathology of dystrophic calcification and heterotopic ossification are well described, the molecular mechanisms that lead to their development and resolution are unknown.

Preclinical models of soft tissue calcification are integral for development of therapeutics that prevent deposition of or promote the resorption of soft tissue calcifications [5–7]. However, the incidence and ultimate fate of soft tissue calcifications in these models is often heterogeneous. Most methods used to quantify the extent of soft tissue calcification, such as histologic analysis or ex vivo micro-computed tomography (μCT), require sacrifice of the animal. Because of the variability between samples and endpoint output measurements, a large number of animals are typically necessary for adequate statistical power to determine the effect of an experimental intervention (i.e., genetic or pharmacologic manipulation) on the incidence or resorption of soft tissue calcification. Therefore, longitudinal quantification of soft tissue calcification would be advantageous in reducing the number of animals required for these types of studies as well as to allow for evaluation of therapeutic intervention of already established soft tissue calcification.

In vivo μCT has been used to quantify heterotopic ossification over time in a murine burn/Achilles tenotomy model[8]. The advantage of in vivo μCT over radiographic imaging in this setting is the increased spatial resolution. Since, soft tissue calcifications in this model are juxtaposed to the tibia, three dimensional imaging (i.e. μCT) is required to delineate the pathologic areas of mineralization from adjacent bone. Despite the potential utility of this method in vivo μCT is more expensive, time consuming, and exposes mice to markedly more radiation than single plane radiography (Table 1) [9].

**Table 1:** Cost, Time, and Radiation Exposure Analysis.

Cost Per Image^1^	$130/Hour	$50/Hour
Time Per Image^2^	~1200 Seconds/ Leg	4 Seconds/ Leg
Radiation Per Image	171–500 mGy^3^	1.4–16.5 mGy^4^

^1^Instutional cost at Vanderbilt University as of May 2016

^2^Scan time necessary to image a single leg of a ~20g mouse

^3^Approximate radiation exposure for a 48 micro resolution image. Variation based on the exact scanning settings and instrument used.

^4^Adverage radiation exposure measured from two independent Faxitron LX-60 cabinets, 20.9cm from x-ray source, 4 seconds at 35 kV, May 2016

As an alternative to the Achilles tenotomy injury, in which injury to muscle is caused by unopposed contracture leading to muscle migration and ischemia [7], other investigators have used cardiotoxin (CTX) to induce muscle injury. CTX instigates muscle fiber depolarization that leads to skeletal muscle death; however, muscle resident stem cells are unaffected, thereby allowing for muscle regeneration following injury [9–11]. Finally, like the Achilles tenotomy injury, CTX-induced muscle injury has also been demonstrated to reliably results in soft tissue calcification [11, 12].

Here, we demonstrate that CTX injection into the posterior compartment of the lower extremity results in formation of soft tissue calcification away from adjacent bones, which allows for serial detection using single plane radiography. Subsequently, we developed and validated a novel, ordinal Soft Tissue Calcification Scoring System (STiCSS) by which the extent of soft tissue calcification can be reliably quantified from digital radiographic images without necessitating the sacrifice of the animal.

## Methods

All animal procedures in this protocol were approved by the Vanderbilt Institutional Animal Care and Use Committee (M/13/099 and M/15/024).

### Murine Models of Soft Tissue Calcification

#### Cardiotoxin-Induced Muscle Injury in Mice Possessing a Genetic Susceptibility for Soft Tissue Calcification

Following adequate anesthesia with Isoflurane, muscle injury to provoke soft tissue calcification was induced by an intramuscular injection of 40μL of 10nM CTX (Accurate Chemical and Scientific Corp, Westbury, NY) into the posterior compartment of the lower leg with a lateral approach using a 28.5G, 0.5ml, insulin syringe. The lower leg of a mouse is divided into two anatomic compartments: the superficial compartment and the deep tissue compartment. Injections were administered primarily in the superficial compartment and within the gastrocnemius and soleus muscles. Studies were conducted using C57B6J (Jackson Lab, Bar Harbor, ME) mice treated with increasing doses of an antisense oligonucleotide (ASO) targeted to plasminogen (IONIS Pharmaceuticals, Carlsbad, CA), a protein previously demonstrated by our lab to protect soft tissue from calcification following injury [13, 14]. Both the right and left posterior compartment muscles of the lower extremity were injured. See S1 Video for a demonstration of the CTX injury-induced soft tissue calcification model.

#### Cardiotoxin-Induced Muscle Injury in Mice Following a Burn Injury

The burn/Achilles tenotomy model previously reported [7] was modified by replacing the tenotomy injury with a CTX injection as described above. All burn studies were conducted in 6-week-old male wild-type C57B6J mice (Jackson Lab, Bar Harbor, ME), with no genetic or ASO-induced predisposition for developing soft tissue calcification, weighing 20–25g as previously described [15–17]. Briefly, prior to adequate anesthesia with Isoflurane, mice received a subcutaneous injection of buprenorphine (0.5-mg/kg) 30 minutes prior to the burn procedure. Following adequate anesthesia with Isoflurane, muscle injury was accomplished by intramuscular injection of CTX into the posterior compartment of the lower extremity as described above. Dorsal hair was then removed and 1ml of saline was injected subcutaneously along the posterior elements of the spine to create a physical buffer from the burn in order to prevent spinal cord injury. The mouse was then placed in a heat-resistant template with the exposed dorsum positioned in the cutout of the template. The template was partially submerged in a 100°C water bath for 10 seconds to create a full-thickness cutaneous burn covering 30% of the body surface area. The mouse was then dried with absorbent bench paper and given 2ml of intraperitoneal resuscitation with lactated Ringer’s solution. Negative control mice received CTX muscle injury without concomitant burn injury.

#### Animal Care and Welfare

Humane endpoints were in place to euthanize any animal that was in pain, unable to eat or drink, experiences wound dehiscence or infection, or lost > 20% of its original body weight. Throughout all investigations, no animals became ill or necessitated sacrifice prior to the designated experimental endpoint.

#### Methods for Monitoring Animals

The physical condition of the mice was monitored continuously following all procedures in which mice were placed under anesthesia until recovery as defined by awakening of the animal, observation of normal movement, and returning to normal eating and drinking behavior.

Mice that underwent a cardiotoxin injury were monitored for visible signs of discomfort or pain once per day for three days following injury. After the first three days, mice were then monitored weekly and weighed at the time of radiographic analysis. Weight measurements were obtained to confirm that mice were not losing >20% of their original body weight, thereby necessitating sacrifice and removal from the study.

Mice that underwent a burn injury with or without a cardiotoxin muscle injury were monitored for visible signs of discomfort or pain twice daily for the first 48 hours after cutaneous burn. After the first 48 hours, mice were monitored daily and weighed weekly at the time of radiographic analysis to confirm that mice were not losing >20% of their original body weight, thereby necessitating sacrifice and removal from the study.

#### Methods to Minimize Potential Suffering

All mice in the study were injured under the influence of an anesthetic (isoflurane). Mice that received a cardiotoxin injury alone did not receive any additional analgesia following injury. Mice that were burned with or without a cardiotoxin muscle injury were administered Buprenorphine at 0.05–0.1 mg/kg of body weight subcutaneously 30 minutes prior to the burn and every 12 hours after the burn for 48 hours. Following the first 48 hours, if a mouse was found to be in visible discomfort or pain following the burn injury, additional Buprenorphine was administered as needed.

#### Methods of Euthanasia

All mice in this study were euthanatized by carbon dioxide inhalation followed by cervical dislocation.

### Analysis of Soft Tissue Calcification Following Injury

#### Radiographic Imaging

Seven days following muscle injury, digital radiographs (Faxitron, Tucson, AZ) of the lower extremity were acquired. Following adequate anesthesia, mice were placed in the prone position with hips in abduction, allowing for external rotation of the leg by placing the tibia in a reproducible lateral position. Single plane lateral radiographic images were collected at an exposure of 4 seconds at 35 kV and saved as a DICOM (.dcm) files for image processing. See S2 Video for a demonstration of radiographic imaging and production of digital radiographs.

#### Post-Image Processing

**Fig 1:**
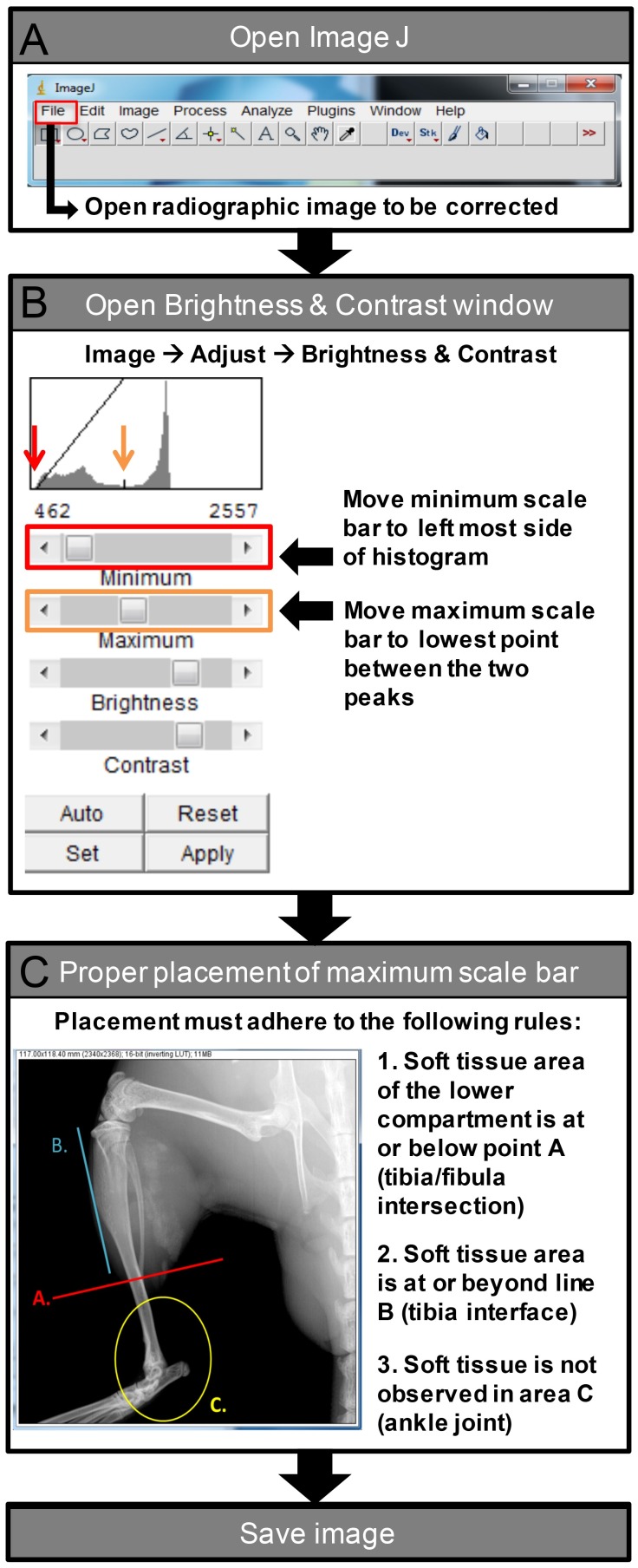
Flow Diagram of Post-Image Processing. Prior to quantification, all digital radiographs underwent post-image processing to ensure appropriate resolution and contrast settings to allow for comparisons between images. The flow diagram demonstrates the stepwise procedure for processing images with ImageJ.

Prior to quantification, all digital radiographs underwent post-image processing to ensure appropriate resolution and contrast settings to allow for comparisons between images. DICOM files were adjusted using ImageJ software (National Institute of Health; http://imagej.nih.gov/ij/) (Fig 1A). Minimum and maximum scale bars were adjusted using the Brightness & Contrast editing tool. The minimum scale cursor was then moved to the leftmost edge of the displayed histogram (Fig 1B) while the maximum scale position was determined following the “ABC” rules (defined in Fig 1C). Proper placement of the maximum scale bar was achieved when the soft tissue area of the lower compartment was at or below line A, at or beyond line B, and was not observed in area C. After the maximum scale bar was properly set, the radiographic image was saved for subsequent scoring [see below].

#### Soft Tissue Calcification Scoring System (STiCSS)

**Fig 2:**
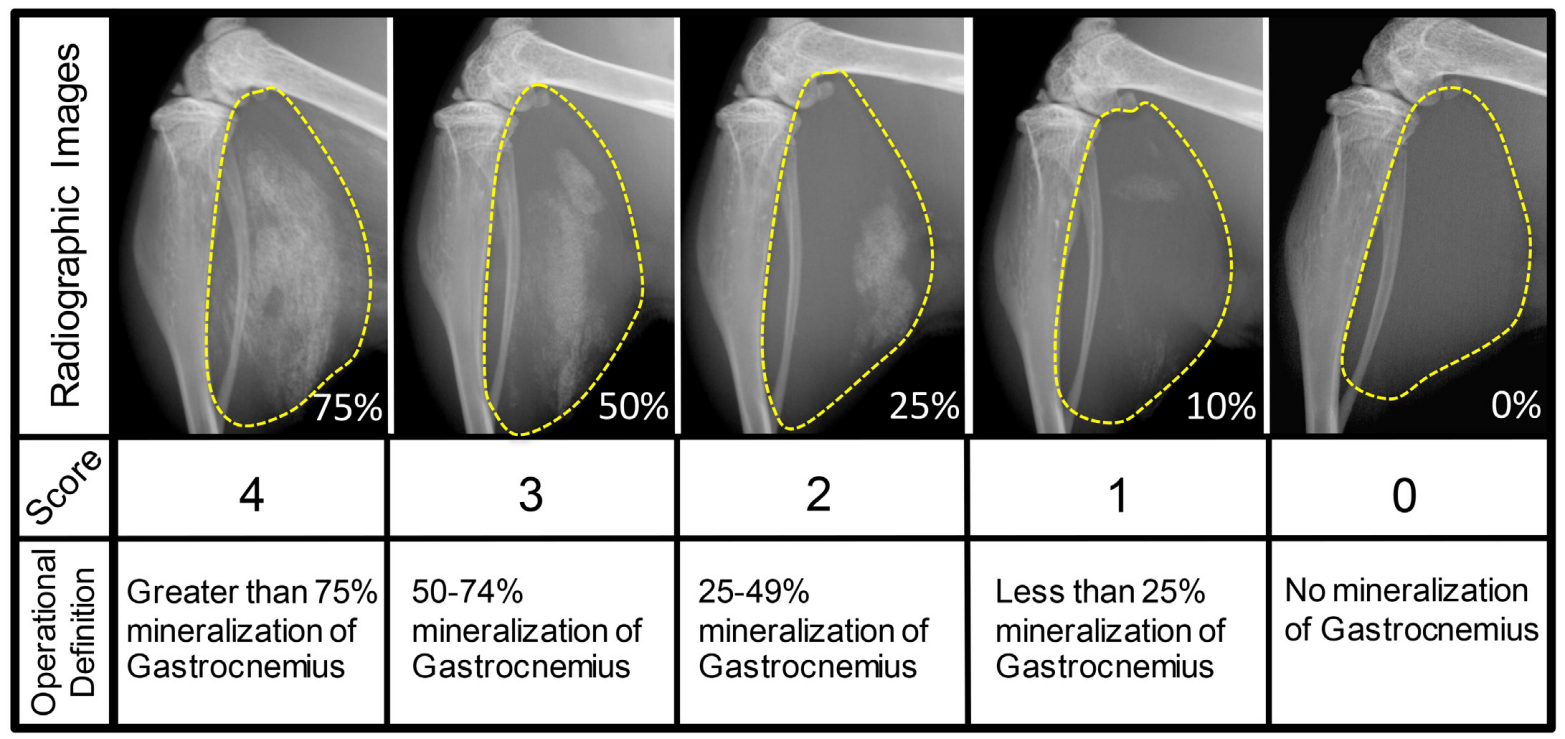
Soft Tissue Calcification Scoring System (STiCSS). STiCSS is an ordinal scale [0–4] developed for quantifying the varying degrees of soft tissue calcification from radiographic images of the lower extremity. Representative images of each STiCSS score are provided along with the operational definition designated to each score. Yellow dotted lines outline the area of interest for soft tissue calcification (the posterior compartment of the lower extremity), while the listed percentages correlate to the extent of soft tissue calcification within each sample image.

Processed digital radiographs were randomized and blinded for quantification. To quantify and statistically evaluate the extent of soft tissue calcification observed in digital radiographs, an ordinal scale (0–4) was formulated according to the varying degrees of soft tissue calcification. The operational definitions of each score are based on the percentage area of soft tissue calcification observed in the posterior compartment of the lower extremity: 0 (0%), 1 (1–25%), 2 (25–49%), 3 (50–75%) and 4 (>75%) (Fig 2). Use of this scale and scoring methods are elaborated in a training module (S3 Video).

#### *Ex vivo* μCT Quantification of Soft Tissue Calcification

μCT images were acquired (μCT40, Scanco Medical AG, Bassersdorf, Switzerland) of injured hindlimbs at 55kVp, 145uA, 200ms integration, 500 projections per 180° rotation, with a 20μm isotropic voxel size following sacrifice. After reconstruction, a volume of interest [18] was selected comprising the region of soft tissue calcification within the posterior compartment of the lower extremity. Mineralized tissue within the VOI was segmented from soft tissue using a threshold of 220 per thousand (or 450.7mgHA/cm^3^), a Gaussian noise filter of 0.2, and support of 1. Mineral volume was calculated using the Scanco evaluation software.

#### Statistical Analysis

To determine the precision and reproducibility of this method, multiple individuals served as adjusters (individual who performed the post-imaging processing; N = 4) and/or observers (individuals who scored previously processed radiographic images using the STiCSS; N = 6).

#### Precision Analysis of Post-Image Processing and Scoring with the STiCSS

For all precision analysis, weighted kappa statistics were calculated for both intra-observer and inter-observer analyses. Kappa values were interpreted using the Landis and Koch criteria as follows: κ <0, less than chance agreement; 0.01–0.20, slight agreement; 0.21–0.40, fair agreement; 0.41–0.60, moderate agreement; 0.61–0.80, substantial agreement; and 0.81–0.99, almost perfect agreement [19]. All kappa statistics were calculated with SAS for Windows 9.4 (SAS Institute, Cary, NC).

#### Intra-observer Error Analysis of Post-Image Processing

To assess the variability of digital radiographic post-image processing, four adjusters were trained as outlined above to process the same set of 40 blinded images with varying degrees of soft tissue calcification within the posterior compartment of the lower extremity following CTX muscle injury. Six individual scorers then evaluated all 160 radiographic images obtained from the four adjusters (i.e. each original image was scored four times- one replicate per adjuster) and the intra-observer agreement between adjusters was assessed.

#### Intra-observer Error Analysis of Image Scoring with the STiCSS

To assess intra-observer variability in the STiCSS, six researchers scored the same set of 40 blinded images twice, with more than one month between scoring sessions. Scores for each image were collected and analyzed using kappa statistics.

#### Inter-observer Error Analysis of Image Scoring with the STiCSS

To assess inter-observer variability in the STiCSS, 160 total images were scored by six individual scorers. Scores for each image were collected and analyzed using kappa statistics.

#### Statistical Correlation of STiCSS Scores and *ex vivo* μCT Quantification

To validate our method as a robust tool for quantifying soft tissue calcification, we compared STiCSS scores to mineral volume measurements obtained from *ex vivo* μCT analysis (threshold 450.7mgHA/cm^3^) as described above. Twenty-eight images with varying degrees of soft tissue calcification were analyzed by both quantitative μCT and the STiCSS to determine the correlation between these two quantification modalities. Correlation was evaluated using a non-parametric Spearman correlation coefficient. Statistical analyses were performed with GraphPad Prism (v6, GraphPad Software, La Jolla, CA).

#### Statistical Evaluation of STiCSS Results

Radiographs were quantified using the STiCSS and scores were individually plotted for each leg with the median and interquartile range. Differences between study groups were assessed using a proportional odds model with random effect. This statistical model takes into account the correlation between the left and the right legs of a single mouse, thereby statistically allowing for inclusion of both legs in our analysis. However, if the number of mice was low or if some of the ordinal categories had very few frequencies, the proportional odds model with random effect failed to fit the data well. Under these circumstances, the Mann-Whitney Rank Test (i.e. a Wilcoxon Rank Sum Test) was used with the understanding that this method fails to account for within-mouse correlation.

#### Sample Size Calculation for the Cardiotoxin-Induced Muscle Injury in Mice with a Burn Injury

The required sample sizes necessary to discern a 25%, 50%, 75%, and 100% differences in soft tissue calcification, as measured by the mean STiCSS score in the Burn/CTX-induced muscle injury models of soft tissue calcification, were determined given β = 0.80 and α = 0.05. While the STiCSS is an ordinal variable, for analysis of the sample size, we used this score as a continuous response variable from matched pairs of study subjects for determination of sample size. All sample size calculations analyses have been by conducted with the PS Power and Sample Size Calculation Program (v3.0, Vanderbilt University, Nashville, TN).

#### Histological Analysis and Staining Procedures

Injured hind limbs were fixed in 10% Neutral buffered formalin for 24 to 72 hours. All samples were processed in graded series of ethanol, cleared, and embedded in paraffin prior to sectioning. 6μm sections were cut and stained as described below.

#### Hematoxylin and Eosin (H&E)

Deparaffinized sections were stained in Gills 3 hematoxylin solution for 5 minutes. Slides were then rinsed in tap water for 10 minutes followed by eosin staining for 2 minutes. Finally, slides were dehydrated and cleared in xylene before mounting with Permount.

#### Von Kossa

Deparaffinized sections were rinsed with distilled water and exposed to 1% silver nitrate solution under ultraviolet light for 30 minutes to develop black staining of mineralization. Slides were then counterstained with Fast green for 5 minutes, dehydrated, and cleared in xylene before mounting with Permount.

## Results

### Precision Analysis of Post-Image Processing

Intra-observer error analysis of post-image process indicated that there were no significant differences among the adjusters performing the post-image processing. Adjusters were in moderate to almost perfect agreement (weighted κ 0.78 to 0.96) when images were scored by 6 individual observers, indicating that the post-image processing method was reliable (Table 2). Additionally, of the 40 individual images that underwent post-image processing, 39/40 (97.5%) demonstrated no significant difference in STiCSS score (as measured by ANOVA), thereby further demonstrating good agreement between adjusters. Together, these findings indicated that the post-image processing method reliably standardizes digital radiographic images for subsequent scoring without significant differences.

**Table 2:** Intra-observer analysis of Post-Image Processing.

Observer A				Weighted Kappa Range
Adjuster	B	C	D	Observer A
A	0.89 [0.81, 0.98]	0.96 [0.91, 1.00]	0.96 [0.91, 1.00]	0.89–0.96
B		0.89 [0.80, 0.98]	0.93 [0.86, 0.99]	
C			0.96 [0.91, 1.00]	
*Observer B*				
Adjuster	B	C	D	Observer B
A	0.92 [0.85, 0.99]	0.85 [0.75, 0.96]	0.90 [0.82, 0.98]	0.82–0.92
B		0.84 [0.74, 0.93]	0.88 [0.80, 0.97]	
C			0.82 [0.71, 0.93]	
*Observer C*				
Adjuster	B	C	D	Observer C
A	0.95 [0.89, 1.00]	0.89 [0.81, 0.98]	0.93 [0.86, 0.99]	0.89–0.95
B		0.95 [0.89, 1.00]	0.95 [0.89, 1.00]	
C			0.93 [0.86, 0.99]	
*Observer D*				
Adjuster	B	C	D	Observer D
A	0.78 [0.67, 0.90]	0.81 [0.70, 0.92]	0.82 [0.72, 0.93]	0.78–0.88
B		0.84 [0.75, 0.94]	0.86 [0.76, 0.95]	
C			0.88 [0.79, 0.98]	
*Observer E*				
Adjuster	B	C	D	Observer E
A	0.85 [0.75, 0.95]	0.93 [0.87, 0.99]	0.85 [0.75, 0.94]	0.83–0.93
B		0.92 [0.83, 1.00]	0.83 [0.71, 0.96]	
C			0.88 [0.80, 0.97]	
*Observer F*				
Adjuster	B	C	D	Observer F
A	0.94 [0.88, 1.00]	0.92 [0.85, 0.99]	0.92 [0.85, 0.99]	0.92–0.94
B		0.94 [0.88, 1.00]	0.94 [0.88, 1.00]	
C			0.92 [0.85, 0.99]	

N = 40 images per Adjusters

### Precision Analysis of the STiCSS

When the same set of 38 blinded radiographic images was scored by the same observer with more than one month between scoring sessions, weighted kappa statistics ranged from 0.88–0.93, showing substantial to almost perfect intra-observer agreement between the two scoring sessions. These results indicated that the variation in soft tissue calcification scoring by each individual observer was minimal (Table 3).

**Table 3:** Intra-observer Error on STiCSS.

Observer	Weighted Kappa	95% CI
A	0.92	0.84, 0.99
B	0.91	0.94, 0.99
C	0.93	0.86, 0.99
D	0.88	0.79, 0.96
E	0.91	0.83, 0.99
F	0.90	0.82, 0.98

N = 38 individual blinded images

Given the minimal intra-observer error, we then determined the agreement between 6 individual observers to assess the inter-observer variability in the STiCSS. When 160 blinded images were scored, weight kappa statistics ranged from 0.73 to 0.90, thereby demonstrating moderate to almost perfect agreement between observers (Table 4). This demonstrates that the STiCSS is a statistically valid and reproducible method for scoring soft tissue calcification within the posterior compartment of the lower extremity following CTX muscle injury without necessitating the sacrifice of the animal.

**Table 4:** Inter-observer Analysis of the STiCSS.

Adjuster A							Weighted Kappa Range
Observer	A	B	C	D	E	F	Adjuster A
A		0.78	0.84	0.74	0.86	0.74	0.74–0.88
B			0.86	0.80	0.78	0.83	
C				0.86	0.88	0.86	
D					0.84	0.82	
E						0.77	
F							
*Adjuster B*							
Observer	A	B	C	D	E	F	Adjuster B
A		0.77	0.89	0.73	0.78	0.74	0.73–0.89
B			0.83	0.81	0.79	0.75	
C				0.76	0.78	0.81	
D					0.76	0.83	
E						0.80	
F							
*Adjuster C*							
Observer	A	B	C	D	E	F	Adjuster C
A		0.76	0.84	0.76	0.83	0.80	0.76–0.85
B			0.82	0.77	0.80	0.77	
C				0.78	0.85	0.82	
D					0.80	0.77	
E						0.84	
F							
*Adjuster D*							
Observer	A	B	C	D	E	F	Adjuster D
A		0.77	0.84	0.77	0.77	0.73	
B			0.90	0.79	0.90	0.82	
C				0.76	0.86	0.82	0.73–0.90
D					0.83	0.78	
E						0.79	
F							

N = 160 images scored per Observer (40 per Adjuster)

### Correlation of the STiCSS to *ex vivo* μCT Quantification

**Fig 3:**
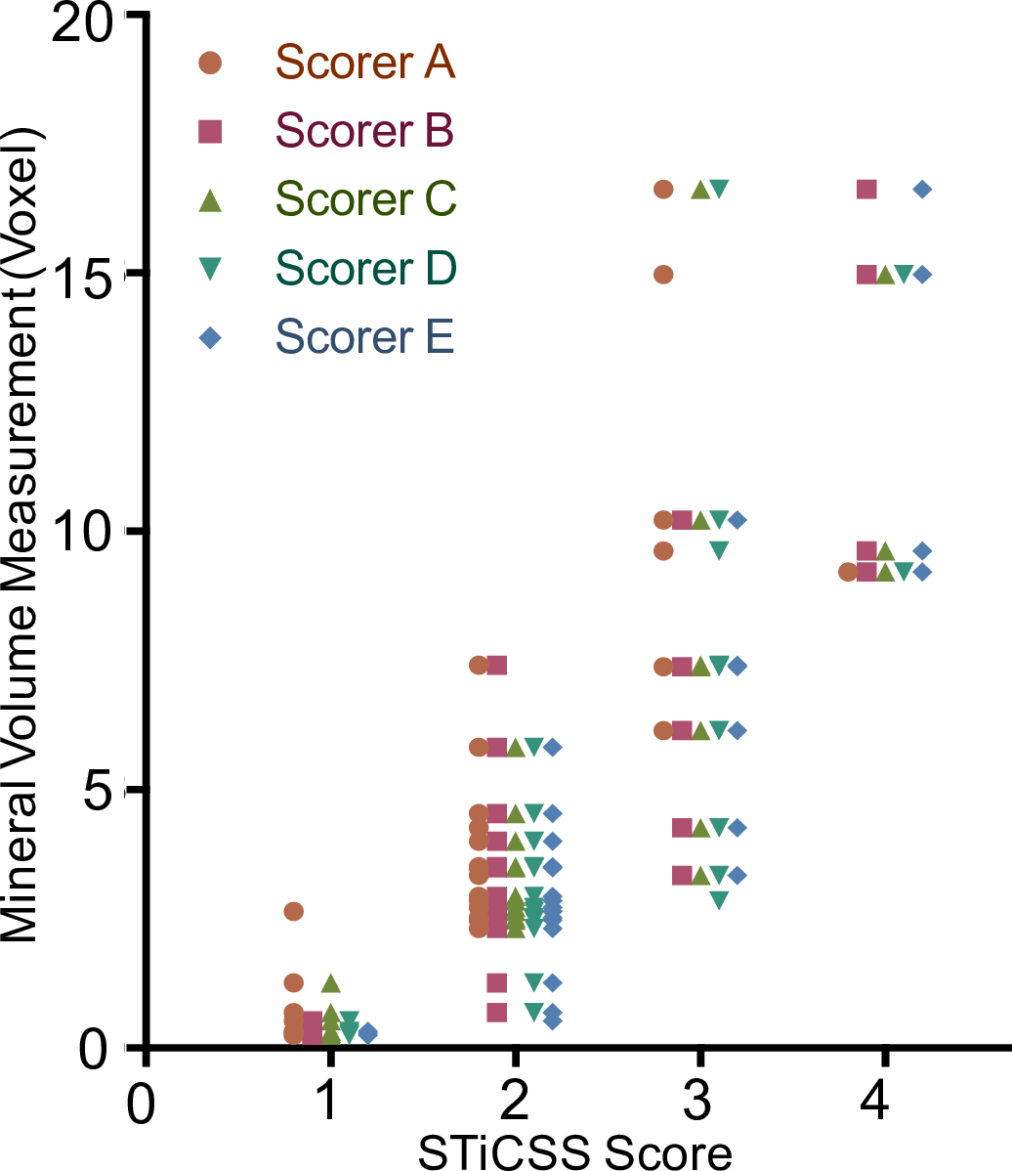
Comparison of STiCSS Score and Mineral Volume Determined by *ex vivo* μCT. STiCSS scores correlated with mineral volume measurement determined by *ex vivo* μCT at a threshold of 450.7mgHA/cm^3^. Correlation was examined using scores from 5 independent observers (A-E) who individually scored 28 images using the STiCSS scale. Scores were then individually plotted against mineral volume measurements obtained by *ex vivo* μCT from the same samples.

Many previous investigations have relied upon μCT to accurately quantify soft tissue calcification. We found that the STiCSS scores among 5 scorers and mineral volume measurements from μCT (threshold 450.7 mgHA/cm^3^) were strongly correlated (Spearman’s r = 0.83 to 0.89, p<0.0001) (Fig 3 and Table 5). Therefore, the STiCSS scoring is consistent with soft tissue calcification quantification by end-point μCT analysis at a threshold of 450.7 mgHA/cm^3^, and thereby represents an affordable, alternative, non-endpoint analysis for quantification of soft tissue calcification within the posterior compartment of the lower extremity.

**Table 5:** Spearman Correlation of the STiCSS and μCT measurements.

Observer	R	95% CI	P
A	0.88	0.76 to 0.95	<0.0001
B	0.84	0.68 to 0.93	<0.0001
C	0.89	0.78 to 0.95	<0.0001
D	0.83	0.66 to 0.92	<0.0001
E	0.86	0.72 to 0.94	<0.0001

N = 28 XY pairs per scorer

### Quantification of Soft Tissue Calcification in the Murine Burn/CTX-Induced Soft Tissue Calcification Model with the STiCSS

**Fig 4:**
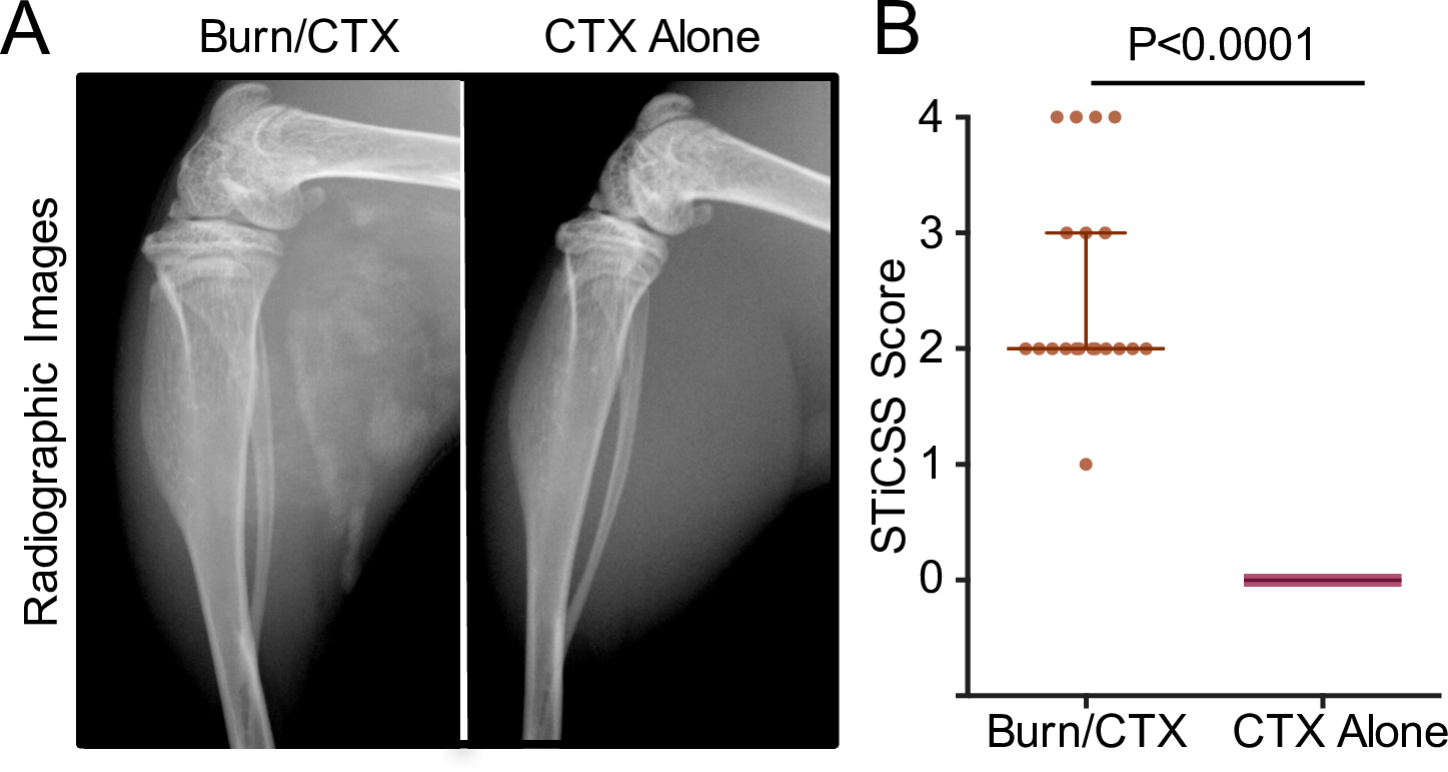
Radiographic Analysis and Quantification of Soft Tissue Calcification Following Burn/CTX Injury. A) Radiographic images of C57BL6 mice that either received a CTX injury alone (N = 8 mice, 16 samples) or a burn injury with a CTX injection (N = 10 mice, 20 samples). B) Graphical representation of radiographic images quantified using the STiCSS. Data represents both the left and right leg of each individual animal. Median and interquartile ranges are shown. Mann-Whitney rank test (p<0.0001, ****) demonstrated significant differences between control (CTX injury alone) and the burn injury group (CTX/Burn Injury).

CTX-induced muscle injury followed by a 30% body surface area, full thickness cutaneous burn resulted in soft tissue calcification separate from the tibia and fibula, thereby allowing for detection by single plane radiography (Fig 4A). Separation from anatomical bone allowed for soft tissue calcification to be detected in radiographic images and be quantified with the STCSS (Fig 4B). Together, these findings demonstrate that we successfully validated the methodology required to quantify soft tissue calcification in a CTX injury model, without sacrifice, using an ordinal scoring system.

### Sample Size Calculation

Sample size calculations for the number of mice needed per group necessary to discriminate 25%, 50%, 75%, and 100% differences in STiCSS scores 7 days following burn/CTX-induced muscle injury are shown in Table 6.

**Table 6:** Sample Size Calculator.

Burn/CTX Injury	
Mean	2.50
SD	0.889
Sample Size Calculations:	
25% Difference	18 mice per group
50% Difference	6 mice per group
75% Difference	4 mice per group
100% Difference	3 mice per group

### Use of STiCSS in Quantifying Dystrophic Calcification and Heterotopic Ossification

**Fig 5:**
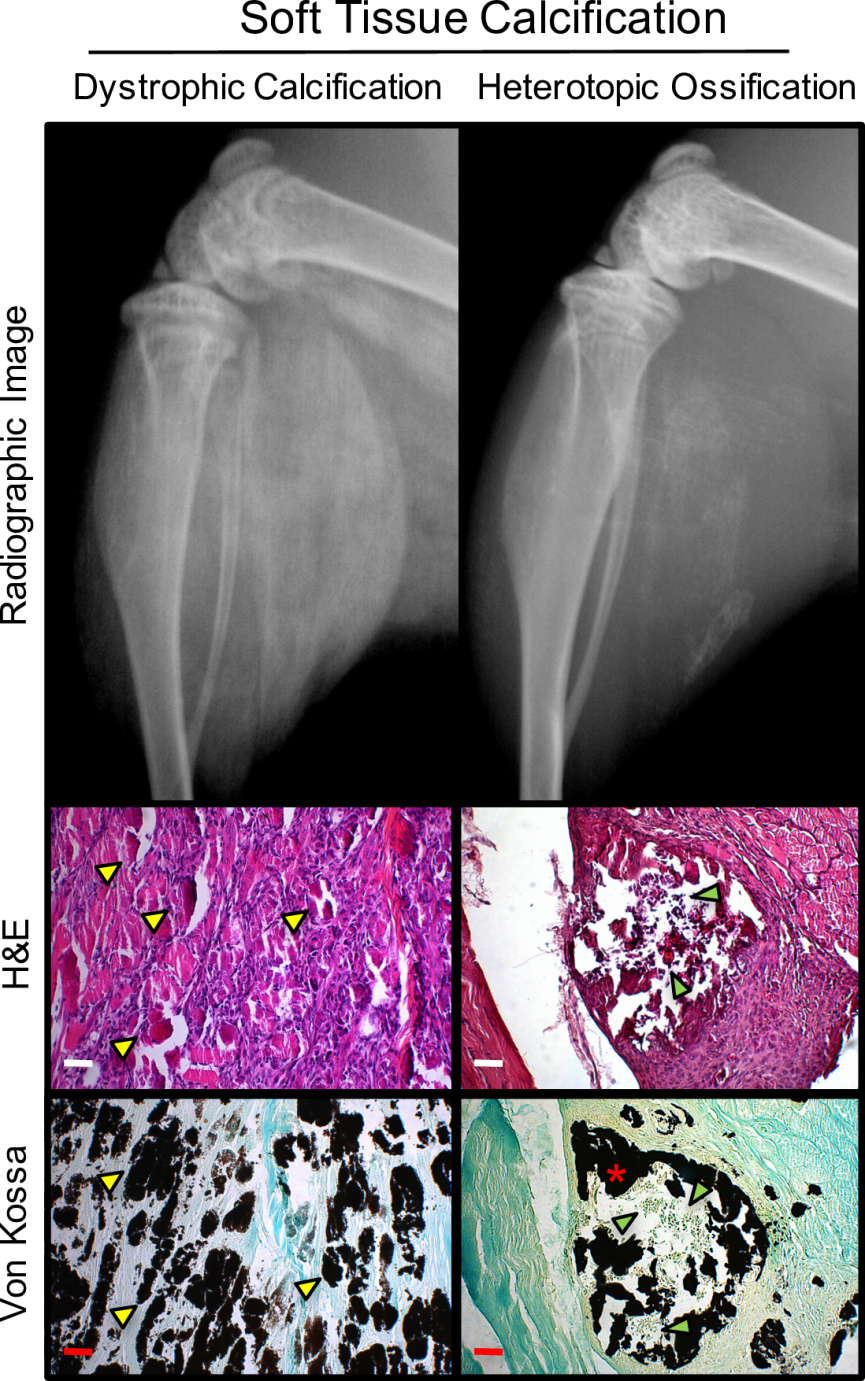
Dystrophic Calcification and Heterotopic Ossification Are Histologically Distinct Yet Radiographically Equivalent. Radiographic and histological images of mice that received a CTX muscle injury and developed dystrophic calcification or heterotopic ossification within the posterior compartment of the lower extremity. Radiographically, dystrophic calcification and heterotopic ossification, two states of soft tissue calcification, are indistinguishable. Nevertheless, the STiCSS can be applied to both processes even though the precise state of mineralization cannot be determined. Histologically, dystrophic calcification and heterotopic ossification can be easily discriminated by their distinct histological characteristics apparent by H&E and Von Kossa (stain for mineralized tissue) staining. Dystrophic calcification is defined histologically by the presence of amorphous, unorganized, calcium phosphate crystals interspersed with necrotic debris at the site of tissue injury (yellow arrow heads). Heterotopic ossification is characterized by mineralized mature bone (red asterisk), which may be associated with a central medullary cavity with intratrabecular hematopoiesis (green arrow heads).

Soft tissue calcification includes dystrophic calcification and heterotopic ossification. Although these processes are histologically distinct, they are radiographically equivalent by STiCSS (Fig 5). The STiCSS represented a reliable method for quantifying the amount of either histological form of soft tissue calcification, but cannot discriminate between them.

## Discussion

The focus of this study was to develop a time and cost effective, reproducible method to quantify soft tissue calcification within skeletal muscle following a CTX injury. Through the induction of a localized muscular injury with CTX injection of the posterior compartment of lower extremity, we were able to provoke consistent soft tissue calcifications away from anatomical bones, thereby allowing for detection by single plane radiography. We then validated a post-image processing method and the STiCSS scoring method for quantifying soft tissue calcification in this model. These methods, unlike other end-point analyses for soft tissue calcification (i.e. *ex vivo* μCT and histological analysis), does not necessitate sacrifice of the animal, thus permitting longitudinal quantification and reduction of the cost of *in vivo* studies. As a means to further lower the costs of this method, all post-image processing methods were performed using freely available software maintained by the National Institute of Health. Thus, radiographic imaging and the STiCSS represent a new, cost-effective, high-throughput, alternative method for longitudinal quantification of soft tissue calcification.

Although *in vivo* μCT can be employed to quantify soft tissue calcification in longitudinal study designs, our methods are relatively inexpensive, less time-consuming, and can be applied more universally in the field of soft tissue calcification, since radiographic imaging is a more widely available imaging platform (Table 1). In addition to the aforementioned considerations, when selecting a method for longitudinal quantification of soft tissue calcification, the amount of radiation exposure during the imaging process is potentially an unknown cofounding variable. Numerous clinical reports have supported the use of radiation as a treatment for reducing soft tissue calcification, specifically heterotopic ossification [20–23]. Consequently, repeated use of this imaging modality may potentially and unknowingly alter the biological processes of soft tissue calcification and experimental results when measured repeatedly. Though the dose of radiation obtained through therapy is much greater than either imaging modality, currently it remains unclear what effect radiation obtained by *in vivo* μCT, if any, may have on soft tissue calcification [24]. Therefore, we proposed that use of radiographic imaging and the STiCSS may be a preferred method for longitudinal soft tissue calcification quantification as the radiation exposure is markedly less (Table 1).

While the STiCSS provides many advantages for longitudinal quantification of soft tissue calcification, one drawback of these methods is the inability of single plane radiography to distinguish between the state of soft tissue calcification, specifically dystrophic calcification and heterotopic ossification. Histologically, dystrophic calcification and heterotopic ossification can be easily distinguished: whereas the former is characterized by islands of disorganized calcium phosphate crystals interspersed with necrotic debris, the latter shows mature bone tissue. Thus, μCT (either *ex vivo* or *in vivo* analysis) or histological analysis on a subset of samples is still necessary to assess the pathological state of soft tissue calcification. Given the pathologic complexity, variable fates of mineralization, and the dynamic nature of this process, murine models which phenocopy the different states of soft tissue calcification following injury and longitudinal quantification methods are both essential. Together, clinically relevant models and validate longitudinal quantification method can be used to delineate the molecular mechanisms resulting in the development and maturation of soft tissue calcifications, as well as to develop and test novel therapeutic strategies. The STiCSS has been demonstrated to reliably quantify the extent of soft tissue calcification, independent of the histopathological state of the mineralization. Therefore, we propose the STiCSS as an effective method to measure various forms of soft tissue calcification.

Due to the ordinal nature of data obtained from the STiCSS as compared to continuous data obtained through μCT quantification, some clustering of the data points is anticipated (Fig 3), since one ordinal score will cover a range of continuous values. Nevertheless, all observers who employed the STiCSS to quantify radiographic images were in good correlation with the values obtained from μCT (Table 5) and were able to reliably score images resulting in substantial agreement with minimal intra/inter observer error.

While this method by no means replaces the use of μCT or histological analysis for determining the pathological state of soft tissue calcification, it does provide an alternative, cost-effective method for longitudinal soft tissue calcification quantification other than *in vivo* μCT. Finally, while the STiCSS is validated for detection of soft tissue calcification in the posterior compartment of the lower extremity, we surmise that due to the robustness of our precision analysis, these methods will be translatable to other trauma-induced soft tissue calcification models as well as other anatomical sites.

## Supporting Information

S1 Video Demonstration of Cardiotoxin-induced Muscle Injury. (MP4)

S2 Video Demonstration of Radiographic Imaging. (MP4)

S3 Video Training Video for Use of the STiCSS. (MP4)
